# Diagnosis and Treatment Pathway for Eosinophilic Granulomatosis with Polyangiitis in the Allergy Department

**DOI:** 10.3390/diagnostics16111734

**Published:** 2026-06-04

**Authors:** Juan Liu, Yingyang Xu, Yuxiang Zhi

**Affiliations:** 1 Beijing Key Laboratory of Precision Medicine for Diagnosis and Treatment of Allergic Diseases, Department of Allergy, Peking Union Medical College Hospital, Chinese Academy of Medical Sciences, Peking Union Medical College, Beijing 100730, China; jane_liujuan@163.com (J.L.); carlxyy@126.com (Y.X.); 2 National Clinical Research Center for Dermatologic and Immunologic Disease (NCRC-DID), Beijing 100730, China

**Keywords:** eosinophilic granulomatosis with polyangiitis, department of allergy, diagnosis, diagnosis and treatment pathway

## Abstract

Eosinophilic granulomatosis with polyangiitis (EGPA) is a rare and heterogeneous autoimmune disease involving multiple organs and systems, characterized by asthma, eosinophilia, and granulomatous or vasculitic involvement of several organs. Most of the initial symptoms include rhinitis, sinusitis, and asthma. The primary consultation departments are typically allergy, otolaryngology, and respiratory. EGPA lacks effective biomarkers for early diagnosis, and most patients have experienced serious multiple organ damage at the time of diagnosis, which makes it more difficult to treat. Therefore, as an allergist, improving the understanding of EGPA, especially the early identification of EGPA in the rhinitis, asthma, or sinusitis stage, will effectively shorten the overall course of EGPA and reduce the mortality and disability rates. Based on this, the author compiled a diagnosis and treatment pathway to outline the clinical manifestations; examination, diagnosis, and treatment pathways; differential diagnosis; disease management; and other aspects of EGPA in the allergic department, aiming to promote early detection, identification, and diagnosis by allergists.

## 1. Introduction

EGPA is a rare anti-neutrophil cytoplasmic antibody (ANCA)-associated systemic vasculitide, which can affect both sexes, with a peak onset age between 30 and 40 years. Although its etiology remains unknown, genetic predisposition [1] and environmental factors [2] are recognized contributors to disease development. Globally reported annual incidence rates range from 0.5 to 4.2 per million, and prevalence is estimated at 10–14 per million [3], although epidemiological data specific to China are lacking. EGPA is a heterogeneous disease that affects multiple organs and can present with a variety of clinical manifestations, frequently non-specific, that often overlap with other pathologies [4], with delays typically ranging from 3 to 9 years from the onset of asthma to the diagnosis of vasculitis [5]. The frequent initial presentation with rhinosinusitis or asthma, coupled with an ANCA positivity rate of only approximately 40% [6], often leads to a diagnosis being established only after the development of multi-organ involvement, complicating treatment and adversely affecting patient prognosis. In a UK retrospective longitudinal study, clinical presentations were predominantly respiratory. The most prevalent comorbidities were asthma (80.6%) and nasal polyposis (32.1%), with allergic rhinitis reported in 16.2% of cases [7]. In our cohort, of the 203 patients (66.6%) who underwent skin testing or allergen-specific IgE evaluation, only 19.7% tested positive for allergen sensitization, which indicated the heterogeneity of atopy among patients with EGPA, and it is worth further attention to the role of atopy in EGPA.

In addition, a retrospective, longitudinal analysis of data from the US Allergy Partners network revealed a substantial disease burden, highlighting the necessity of multidisciplinary collaboration to optimize treatment strategies [8]. Furthermore, patients were treated at the allergy department (48%) [9]. Thus, for Allergists, the early identification of EGPA among patients presenting with refractory asthma accompanied by rhinitis or sinusitis is crucial for prompt intervention and treatment, thereby improving outcomes. The aim of this diagnostic and therapeutic pathway for EGPA in the Allergy department is to enhance allergists’ early recognition and diagnostic capabilities for EGPA.

## 2. Pathogenesis and Triggering Factors

The precise triggers of EGPA remain incompletely understood. Current evidence supports EGPA as a heterogeneous disorder resulting from the interaction of genetic susceptibility, environmental exposures, and dysregulated immune inflammation [10]. Its core pathogenic mechanisms involve abnormalities in both innate and adaptive immunity, including T lymphocytes, B lymphocytes, eosinophils, and neutrophils; HLA-associated genetic backgrounds may also influence disease susceptibility and clinical phenotypes [11,12]. Overall, EGPA is characterized predominantly by type 2 (T2) inflammation and eosinophil-mediated tissue injury, although ANCA-positive and ANCA-negative patients exhibit distinct immunologic and clinical features [13].

### 2.1. T Lymphocytes and Innate Immune Pathways

Multiple immune pathways, including Th1, Th2, and Th17 responses, are activated in EGPA, with eosinophil-mediated inflammatory injury representing a major pathological hallmark [14]. Among these pathways, Th2 immunity is generally considered the principal driver of eosinophilic inflammation. Increased levels of Th2-associated cytokines have been detected in the serum of patients with active EGPA [15,16]. At the same time, elevated expression of Th2 and regulatory transcripts has been observed in bronchoalveolar lavage fluid (BALF) cells [17]. Restricted T-cell receptor repertoires further suggest antigen-driven T-cell activation. Activated Th2 cells secrete cytokines such as IL-3, IL-4, IL-5, IL-10, and IL-13, thereby promoting eosinophil maturation, activation, and persistence of inflammation [10]. Among these cytokines, IL-5 is the key regulator of eosinophil differentiation, maturation, activation, and survival, primarily by inhibiting apoptosis [18]. Because IL-5 receptor (IL-5R) expression is largely restricted to eosinophils, targeting the IL-5/IL-5R axis has become a major therapeutic strategy in EGPA [19].

Th17, Th1, and innate immune pathways also contribute to amplification of inflammation in EGPA. Increased Th17 cells, and reduced regulatory T-cell (Treg) frequencies, and elevated Th17/Treg ratios have been associated with disease activity [20]. Elevated serum IFN-γ levels and detection of Th1 cells in affected tissues suggest that Th1 responses may contribute to granuloma formation and vascular injury. In addition, increased levels of IL-33, thymic stromal lymphopoietin (TSLP) [21], and type 2 innate lymphoid cells (ILC2s) have been identified in active EGPA, indicating that epithelial-derived “alarmins” may promote T2 inflammation through activation of ILC2s and Th2 responses [22]. ILC2s are characterized by high GATA3 expression and production of IL-5 and IL-13, further supporting their role in eosinophil recruitment and maintenance of inflammation [12].

### 2.2. Eosinophils and the IL-5/IL-5R Axis

Eosinophils are among the central effector cells responsible for tissue injury in EGPA. Abnormal eosinophil proliferation, impaired apoptosis, tissue infiltration, and release of cytotoxic granule proteins are closely associated with organ damage [23]. Increased eosinophil infiltration and extracellular protein deposition have been observed in tissues such as skin [24] and endomyocardial specimens [25]. Activated eosinophils release granule proteins, including major basic protein (MBP), eosinophil cationic protein (ECP), eosinophil peroxidase (EPO), and eosinophil-derived neurotoxin (EDN), resulting in direct tissue toxicity [26] and indirect inflammatory injury through recruitment of additional immune cells. Pathological consequences include thrombosis, fibrosis, and allergic inflammation [27]. Eosinophil-derived mediators such as IL-25 may further amplify Th2 responses and correlate with disease activity [28].

Eosinophil chemokines also play important roles in eosinophil recruitment. CCL11, CCL24, and CCL26 can be produced by endothelial cells and T lymphocytes, while IL-4 and IL-13 synergistically enhance their production [29]. Among these chemokines, CCL26 binds to CCR3, which is highly expressed on eosinophils; elevated CCL26 levels have been associated with EGPA disease activity. From the perspective of IL-5 signaling, eosinophils originate in the bone marrow and migrate into inflamed tissues under the influence of IL-5 and chemotactic factors [18]. IL-5 is produced by Th2 cells, ILC2s, mast cells, basophils, and dendritic cells. Although its immunologic effects may extend beyond eosinophils, eosinophils remain the major effector cells. IL-5 promotes eosinophil differentiation, migration, activation, degranulation, and resistance to apoptosis, thereby increasing eosinophil numbers and effector functions in both blood and tissues.

ANCA-negative EGPA is typically characterized by more prominent eosinophil-driven tissue infiltration. Activated eosinophils may form eosinophil extracellular traps (EETs), contributing to endothelial injury, microthrombosis, and fibrosis [30]. Clinically, ANCA-negative disease is more frequently associated with cardiac involvement, pulmonary infiltrates, and gastrointestinal manifestations, while renal involvement may present as membranous nephropathy or interstitial nephritis rather than pauci-immune glomerulonephritis [31]. Furthermore, reports of FIP1L1-PDGFRA-positive EGPA and responses to imatinib suggest that a subset of patients may share tyrosine kinase-related mechanisms with hypereosinophilic syndrome (HES) [32].

### 2.3. B Lymphocytes, ANCA, and Neutrophils

The role of B lymphocytes in EGPA pathogenesis has received increasing attention. Evidence, including the efficacy of anti-CD20 therapy [33], elevated serum IgE and IgE-containing immune complexes, increased IgG4 levels [34,35], and expansion of plasmablasts and follicular helper T cells, suggests that B-cell activation contributes to disease development and progression. ANCA represents another manifestation of B-cell activation, although its pathogenic role in EGPA remains incompletely defined. Experimental and human studies support the ability of MPO-ANCA to induce neutrophil activation and endothelial injury [36]. However, only approximately one-third of EGPA patients are ANCA-positive, a frequency substantially lower than that observed in granulomatosis with polyangiitis (GPA) and microscopic polyangiitis (MPA) [37]. Moreover, although both EGPA and MPA are predominantly associated with MPO-ANCA, their clinical phenotypes differ markedly. These findings suggest that ANCA-related mechanisms in EGPA may be influenced by factors such as epitope specificity, epitope conformation, or epitope masking [11].

ANCA-positive EGPA more commonly exhibits manifestations related to small- and medium-vessel vasculitis. MPO-ANCA interacts with neutrophils, inducing respiratory burst activity and release of proteolytic enzymes and reactive oxygen species, thereby contributing to necrotizing vasculitis and granuloma formation [38]. Clinically, ANCA-positive patients more frequently develop glomerulonephritis, mononeuritis multiplex, purpura, and systemic vasculitic manifestations. In recent years, neutrophil extracellular traps (NETs) have been implicated in the pathogenesis of ANCA-associated vasculitis (AAV) and may serve as sources of ANCA antigens [39]. Enhanced NET formation has been observed in EGPA and correlates with peripheral eosinophil counts [40]. In addition, EETs and eosinophil extracellular trap cell death (EETosis) have been reported to contribute to inflammation and thrombosis in EGPA [30,41].

### 2.4. Genetics

Multiple immunogenetic factors have been associated with EGPA susceptibility and phenotypic heterogeneity. HLA-*DRB107*, *HLA-DRB104*, and HLA-DRB4 have been linked to EGPA development or CD4+ T-cell activation, whereas HLA-*DRB103* and *HLA-DRB113* may confer protective effects [42,43]. Variants in the IL-10 promoter region have also been associated with ANCA-negative EGPA [44]. Genome-wide association studies (GWAS) [1] further demonstrated that ANCA-positive EGPA shares part of its genetic background with MPO-associated AAV through HLA-DQ-related variants, whereas ANCA-negative EGPA appears more strongly associated with variants related to mucosal barrier function and the IL-5/IRF1 pathway. Shared susceptibility loci, including TSLP, BCL2L11, and CDK6, have also been identified in both subsets.

### 2.5. Environmental Triggering Factors

Various environmental factors have been reported as potential triggers or precipitating factors for EGPA, including allergens, infectious pathogens, medications, vaccinations, and desensitization therapies [45]. Reported associations include inhaled allergen exposure, Aspergillus infection, and allergic bronchopulmonary aspergillosis (ABPA) [46,47]; post-COVID-19 onset [48]; and disease emergence following leukotriene receptor antagonists (LTRAs) [49], anti-IgE therapy [50], or certain anti-interleukin therapies [51]. However, most evidence derives from case reports or observational studies, and findings regarding the relationship between LTRA therapy and EGPA risk remain inconsistent [52]. Moreover, systematic allergy testing has shown that fewer than one-third of patients demonstrate clear allergic sensitization [53]. Therefore, environmental and drug-related factors are more appropriately regarded as potential triggers or unmasking factors rather than definitive causes of EGPA.

### 2.6. Summary

In summary, EGPA can be viewed as a complex disorder centered on T2 inflammation and eosinophil-mediated tissue injury, while also involving ANCA-associated vasculitis, B-cell activation, NETs/EETs, genetic predisposition, and environmental exposures. ANCA-negative patients typically exhibit more prominent eosinophilic tissue infiltration and organ damage, whereas ANCA-positive patients more commonly display vasculitic phenotypes. The IL-5/IL-5R axis plays a central role in eosinophil generation, migration, activation, and survival, providing the mechanistic basis for therapies targeting IL-5 or IL-5R. Environmental and drug-related triggers should be interpreted cautiously within the appropriate clinical context.

## 3. Clinical Manifestations

EGPA is a multisystem disease commonly involving the sinuses, lungs, nervous system, heart, gastrointestinal tract, skin, and kidneys. The most frequent symptoms are asthma and rhinosinusitis. The natural history of EGPA is often described in three overlapping phases: a prodromal “allergic” phase, an eosinophilic tissue infiltration phase, and a vasculitic phase [3].

### 3.1. Respiratory System

The primary respiratory manifestations of EGPA include wheezing, cough, rhinitis, nasal polyps, or sinusitis. Asthma is present in 90–100% of patients [54,55,56], typically as the initial symptom, and is often associated with upper airway allergic symptoms and persistent airflow limitation, usually without significant seasonal variation, and tends to worsen over time. These patients are frequently ANCA-negative [57]. Reports indicate that over 40% of EGPA patients have severe or uncontrolled asthma [58]. Approximately 25–30% of EGPA patients have an atopic background [59]. One cohort study found that among the 33.5% of EGPA patients tested for allergens, about two-thirds were sensitized to aeroallergens, primarily house dust mites and grass pollen; these patients often had higher serum total IgE levels and were more likely to present with refractory or severe asthma before progressing to vasculitis [60]. The transition from the prodromal phase to the eosinophilic infiltration phase occurs after a mean duration of approximately 9.3 ± 10.8 years [55]. Therefore, the presence of difficult-to-control or severe asthma characterized by high eosinophilia, recurrent sinusitis with or without nasal polyps, should raise suspicion for early-stage EGPA.

Pulmonary imaging in EGPA shows bilateral consolidation or reticulonodular opacities in 70% of cases, pulmonary infiltrates in 40–50% [3], and airway wall thickening and bronchiectasis in 66% [61]. The 1990 American College of Rheumatology (ACR) criteria listed transient or migratory pulmonary infiltrates as a characteristic radiographic finding for EGPA [62].

### 3.2. Other Systemic Manifestations

**Skin:** Skin involvement occurs in 20–67% of EGPA patients [22] and can be diverse. Cutaneous purpura is considered a specific lesion of EGPA vasculitis.

**Cardiac:** Cardiac involvement occurs in approximately 11–74% of patients [12,63], is more common in ANCA-negative individuals, carries a poor prognosis, and is a leading cause of death in EGPA. Clinical presentations include myocarditis (often with thrombosis), endocarditis, pericarditis, valvulopathy, and coronary arteritis [64,65].

**Gastrointestinal (GI):** GI tract involvement is seen in 24–78% of cases [66], manifesting as abdominal pain (30–91%), diarrhea (45%), or gastrointestinal bleeding (3–9%). Rarely, intestinal infarction and ischemic colitis (1–3%) may occur. Biopsy may reveal eosinophilic infiltration, sometimes accompanied by vasculitis and eosinophilic granulomas [67].

**Nervous System:** Neurological involvement is observed in approximately 42–76% of patients, with mononeuritis multiplex being a classic presentation [68,69]. Central nervous system involvement occurs in about 25% of patients [68]. The 2025 Chinese Multidisciplinary Expert Consensus on the Diagnosis and Management of EGPA recommends excluding EGPA in any asthmatic patient presenting with neurological symptoms [70].

**Renal:** Renal involvement is less common (16.3–35%) in EGPA but is often associated with ANCA positivity [71]. Clinical manifestations primarily include proteinuria and renal insufficiency; interstitial nephritis with eosinophilic infiltration is rare [72].

## 4. Detection Methods

The absence of a definitive biomarker for EGPA diagnosis makes early recognition through meticulous clinical history-taking and targeted auxiliary investigations crucial.

### 4.1. Hematological Parameters

**Peripheral blood eosinophils:** An absolute eosinophil count > 1000/μL and/or a percentage > 10% often suggests active EGPA and is one diagnostic criterion [22]. However, in patients receiving systemic corticosteroids (e.g., for asthma), peripheral eosinophil counts can drop rapidly within days, masking the true baseline level. Therefore, ascertaining pre-treatment eosinophil levels is important. Experts have proposed that EGPA should be considered in patients aged 6 years or older who present with blood eosinophil levels exceeding 1000 cells/µL if untreated, or greater than 500 cells/µL if they have previously received therapies that may affect eosinophil counts [73].

**ANCA:** ANCA is detected in approximately 30–40% of patients, mostly perinuclear ANCA (p-ANCA) and myeloperoxidase-ANCA (MPO-ANCA) specific [37]. ANCA-positive patients tend to have more frequent cardiac, gastrointestinal, and pulmonary involvement, while ANCA-negative patients more commonly exhibit granulomatous manifestations, peripheral neuropathy, and renal disease. A positive ANCA test strongly supports an EGPA diagnosis in a patient with asthma, eosinophilia, sinusitis, and pulmonary infiltrates [74].

**Serum Immunoglobulins:** Total IgE levels are often elevated in EGPA, sometimes higher than in allergic asthma [75]. If allergic diseases are present in the prodromal phase, allergen-specific IgE levels may also be elevated, suggesting potential utility of allergen screening. Serum IgG4 levels are significantly elevated in active EGPA and correlate with the extent of organ involvement, indicating a potential role for IgG4 in disease activity [76].

**Other parameters:** Erythrocyte sedimentation rate (ESR) and C-reactive protein (CRP) are considered indicators of disease activity. Interleukins (IL)-6, IL-8, IL-10, and tumor necrosis factor-alpha (TNF-α) may be elevated in EGPA patients [77]. Some patients may test positive for antinuclear antibodies (ANA) [78]. Cardiac involvement can lead to elevated cardiac enzymes.

### 4.2. Respiratory System Investigations

Elevated eosinophil counts in induced sputum or BALF (often >25%) and the presence of ANCA are significant features [23]. Some serum ANCA-negative patients may test positive for ANCA in induced sputum, potentially serving as a novel diagnostic marker [79]. Sinus CT is recommended for patients with sinusitis or nasal polyps. Pulmonary function tests (PFTs), including spirometry, lung volumes, diffusing capacity, and bronchodilator response, should be routinely performed. EGPA patients may show reversible airflow obstruction, airway hyperresponsiveness, and reduced diffusing capacity. Fractional exhaled nitric oxide (FeNO) can be used to assess response to inhaled corticosteroid therapy. Chest CT findings help distinguish EGPA from refractory asthma and may include tree-in-bud opacities, ground-glass opacities, bronchiectasis, nodules, consolidation, and interstitial changes [70].

### 4.3. Skin

A readily accessible biopsy site; pathology may show granulomas or necrotizing lesions.

### 4.4. Cardiac

Patients with suspected cardiac involvement should undergo echocardiography and electrocardiography. Cardiac magnetic resonance imaging (CMR) showing late gadolinium enhancement suggests myocardial involvement. Endomyocardial biopsy can provide definitive evidence of vasculitis but carries procedural risks.

### 4.5. Gastrointestinal

Endoscopy may be considered for patients with relevant GI symptoms.

### 4.6. Nervous System

Electromyography (EMG) is indicated for peripheral neuropathy; nerve or muscle biopsy may be considered. A brain MRI is recommended for central nervous system symptoms.

### 4.7. Renal

Urinalysis and quantification of urinary protein should be performed routinely. Kidney biopsy is an option if renal involvement is suspected.

## 5. Diagnosis and Differential Diagnosis

### 5.1. Diagnosis

**Figure 1:**
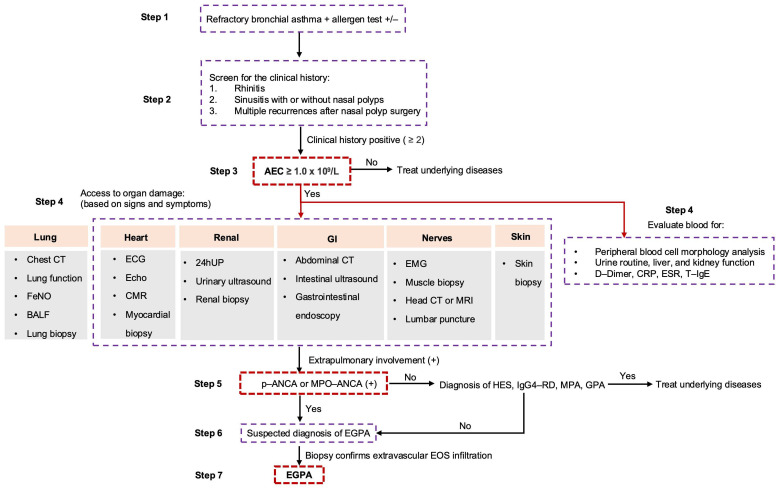
Diagnostic and Treatment Pathway for EGPA in the Allergy Department. AEC: absolute eosinophil count; FeNO: fractional exhaled nitric oxide; BALF: bronchoalveolar lavage fluid; ECG: electrocardiogram; Echo: echocardiography; CMR: cardiac magnetic resonance; 24hUP: 24-h urinary protein; EMG: electromyography; CRP: C-reactive protein; ESR: erythrocyte sedimentation rate; T-IgE: total immunoglobulin E; p-ANCA: perinuclear ANCA; MPO-ANCA: myeloperoxidase-ANCA; EGPA: eosinophilic granulomatosis with polyangiitis; HES: Hypereosinophilic syndrome; GPA: granulomatosis with polyangiitis; MPA: microscopic polyangiitis; IgG4-RD: IgG4-related disease; GI: Gastrointestinal.

There is no universally accepted diagnostic criterion for EGPA. Classification criteria commonly used include the ACR criteria [62] and the 2022 ACR/European Alliance of Associations for Rheumatology (EULAR) criteria [4] (Table 1). Given the increasing rate of early visits by EGPA patients to allergy departments [9], it is essential to establish a diagnostic and treatment pathway aimed at facilitating early recognition of EGPA by allergists. A proposed diagnostic workflow for suspected EGPA patients presenting to the Allergy department is illustrated in Figure 1.

### 5.2. Differential Diagnosis

Key differential diagnoses include wheezing-associated disorders and other hypereosinophilic diseases.

#### 5.2.1. Wheezing-Associated Disorders

**Asthma:** Often has an atopic history, is ANCA-negative, features mild or normal peripheral eosinophil counts, rarely involves other organs, typically has normal diffusing capacity, and chest imaging shows bronchial wall thickening/mucus plugging without migratory infiltrates.

**ABPA** [80,81]**:** Associated with asthma, cystic fibrosis, or tuberculosis history; positive *Aspergillus*-specific IgE or IgG; markedly elevated total IgE (often >1000 IU/mL); no extra-pulmonary organ involvement; and chest CT may show central bronchiectasis or mucoid impaction (finger-in-glove sign).

**Chronic Eosinophilic Pneumonia (CEP)** [82]**:** Also common in asthmatics/atopic individuals, more frequent in women; BALF shows very high eosinophil percentage; may have elevated IgE, anemia, thrombocytopenia; chest CT shows non-migratory peripheral ground-glass opacities; and extra-pulmonary involvement is rare.

**IgG4-Related Disease (IgG4-RD)** [83]**:** Can share features like asthma, rhinitis, or eosinophilia; distinguishing feature is histopathological evidence of dense lymphoplasmacytic infiltrate rich in IgG4^+^ plasma cells and storiform fibrosis.

#### 5.2.2. Hypereosinophilic Diseases

HES [84]: A major differential diagnosis. Clinically, ~40% of HES patients exhibit lung involvement, while only about 11% have asthma. Idiopathic HES is most challenging to distinguish from ANCA-negative EGPA without overt vasculitis; the relative infrequency of asthma and sinusitis in HES may be a clue, but it is not specific.

#### 5.2.3. Other Vasculitides [85]

Includes GPA, MPA, and polyarteritis nodosa (PAN). Key differentiating features are summarized in Table 2.

## 6. Treatment Principles, Strategies, and Follow-Up

### 6.1. Treatment Principles and Strategies

EGPA management should always be multidisciplinary. Treatment is stratified based on disease severity, organ involvement, and activity into induction of remission and maintenance therapy (Table 3) [86]. Active severe EGPA: Defined by life-threatening or major organ-threatening involvement (e.g., cardiac, mesenteric ischemia, alveolar hemorrhage, glomerulonephritis, severe ocular or peripheral neuropathy). Active non-severe EGPA: Lacks life-threatening or major organ manifestations (e.g., asthma, sinusitis, minor systemic symptoms). Given the frequent respiratory manifestations, local therapy for asthma and rhinosinusitis is recommended. The 2025 Chinese EGPA guideline [70] suggests treating asthma according to GINA steps 4–5, recommending high-dose inhaled corticosteroids combined with long-acting bronchodilators, potentially adding leukotriene receptor antagonists.

### 6.2. Biologic Therapies

In recent years, novel targeted biologic therapies have become available, leading Allergists to place greater focus on the type 2 immune response that predominantly drives eosinophilic inflammation. Overall, the EULAR recommendations currently recognize that mepolizumab and rituximab are the only biologic therapies for EGPA [86]. In particular, anti-IL-5 strategies, as evaluated in the MIRRA [19] and MANDARA trials [87], together with data from the French REOVAS study (testing rituximab) and other real-world cohorts, have provided important evidence for the management of relapsing or refractory EGPA.

#### 6.2.1. Anti-IL-5 Therapies

Anti-IL-5 agents, including mepolizumab (an anti-IL-5 monoclonal antibody), reslizumab (a humanized anti-IL-5 antibody), and benralizumab (an anti-IL-5 receptor α chain), have demonstrated efficacy in reducing eosinophil counts, enabling glucocorticoid (GC) dose reduction, increasing remission rates, and lowering relapse rates [88].

Mepolizumab is the first FDA-approved anti-IL-5 monoclonal antibody for EGPA [89]. It is recommended as the preferred adjunctive therapy for the maintenance of remission in patients with relapsing EGPA without organ- or life-threatening manifestations [86]. Long-term data from the MARS study support the favorable safety and efficacy of the 300 mg every 4 weeks regimen [90]. Although this dosage was used in the MIRRA trial [19], real-world evidence from a large retrospective observational study indicates that many patients with EGPA are treated with the asthma-approved dose (100 mg every 4 weeks) [91]. Clinical outcomes appeared broadly comparable between the 100 mg and 300 mg regimens, although a numerically higher proportion of patients receiving 300 mg achieved complete remission at 24 months. Notably, a dose-reduction study in patients in stable remission demonstrated that stepping down from 300 mg to 100 mg every 4 weeks did not lead to worsening of asthma or vasculitis over a median follow-up of 27.5 months [92]. Approximately 50% of patients maintained complete remission without oral GCs, while the remaining patients experienced recurrence of sinonasal symptoms, which were effectively controlled by increasing the mepolizumab dose or optimizing local therapy. Real-world data from Europe, the United States, and Japan further corroborate the findings of the MIRRA trial. At 12 months, approximately 58% of patients achieved complete or partial remission, increasing to 92% at 24 months in cohorts treated with the approved dose (European EGPA cohort study) [91]. Treatment was associated with reduced oral GC use, lower relapse and hospitalization rates, and decreased asthma exacerbations, alongside a reduction in GC-related complications (U.S. studies) [93]. Long-term follow-up data from Japan showed that 36% of patients were able to discontinue oral GCs by weeks 93–96, while only 8% required high-dose therapy [90]. From the patient perspective, mepolizumab also led to rapid and significant improvements in health-related quality of life, as assessed by the AAV-Patient-Reported Outcomes (PROs) questionnaire, and reduced multi-organ disease burden [94,95].

Benralizumab is a humanized monoclonal antibody targeting the interleukin-5 receptor α (IL-5Rα) on eosinophils, inducing antibody-dependent cellular cytotoxicity and rapid eosinophil depletion. It is currently approved by the FDA for severe eosinophilic asthma and EGPA [96]. The phase III MANDARA trial compared benralizumab (30 mg every 4 weeks) with mepolizumab (300 mg every 4 weeks) in adults with relapsing or refractory EGPA receiving standard therapy [87]. Benralizumab demonstrated non-inferiority in remission rates at weeks 36 and 48 (59% vs. 56%; difference 3%, 95% CI—13 to 18). Furthermore, between weeks 48 and 52, the benralizumab group had a lower mean GC dose and a higher proportion of patients achieving complete GC withdrawal. A post hoc analysis of the MANDARA trial showed that benralizumab was associated with higher rates of complete remission (BVAS = 0 and oral GC dose ≤0 mg/day at weeks 36 and 48) and sustained complete remission (achieved at week 48 and maintained through week 52). These findings were supported by a large European real-world comparative study conducted by the European EGPA Study Group, in which both agents were administered at asthma-approved doses [97]. Benralizumab achieved higher complete remission rates at 12 months and was associated with deeper peripheral eosinophil depletion. A meta-analysis of eight studies (including randomized and observational data) confirmed high overall remission rates and a significant reduction in GC dose (estimated effect: −8.25 mg/day) [98].

Reslizumab is a third IL-5-targeting biologic currently approved for asthma. It binds to IL-5 and inhibits eosinophil activation and proliferation. Its use in EGPA has been evaluated in a small phase II open-label study (RITE study, NCT02947945), in which 10 patients received 3 mg/kg every 4 weeks [99]. The treatment was generally well tolerated and associated with a significant reduction in daily oral GC dose. However, 3 patients experienced disease exacerbations, and 1 patient developed a serious adverse event requiring treatment discontinuation. At present, its use in EGPA remains off-label and requires further validation.

A randomized, double-blind, controlled trial comparing depemokimab with mepolizumab is currently ongoing (NCT05263934). Depemokimab is an ultra-long-acting biologic with enhanced IL-5 binding affinity, potentially allowing dosing intervals of up to 6 months. Another investigational agent, SHR-1703 (Jiangsu Hengrui Pharmaceuticals, Liangyungang, China), is a novel humanized IgG1 monoclonal antibody with high IL-5 affinity and prolonged half-life, currently under evaluation in a double-blind, placebo-controlled trial (NCT05583227).

#### 6.2.2. Other Emerging Targeted Therapies Beyond IL-5

Dupilumab (anti-IL-4/IL-13): Has shown efficacy for refractory or recurrent sinonasal symptoms related to EGPA [100,101]. However, its efficacy and safety remain uncertain. A European multicenter retrospective study suggested potential benefits in controlling ENT symptoms, but approximately one-third of patients experienced EGPA flares, often preceded by eosinophilia (observed in 88% of relapses). Therefore, caution is warranted, particularly in patients with uncontrolled systemic disease. A recent small prospective observational study confirmed its efficacy in refractory chronic rhinosinusitis associated with EGPA [102], but again reported a high rate of adverse events, including hypereosinophilia, with some cases requiring treatment discontinuation. Tezepelumab has only been evaluated in small case series in EGPA [103]. Nanzer et al. reported its use in eight patients with refractory EGPA who remained GC-dependent despite anti-IL-5/5R therapy. Improvements in BVAS, reductions in annual relapse rate, and a non-significant decrease in GC dose were observed. Two ongoing randomized trials (RACEMATE: NCT06230354; CROSSING: NCT05583227) are expected to further clarify its role. Omalizumab (anti-IgE): May have a GC-sparing effect in EGPA patients with asthma and/or nasal symptoms [104]. Based on the observation that EGPA patients with atopy often present with more severe uncontrolled asthma yet less severe vasculitis, the positive effects of omalizumab in EGPA may be associated with the atopic status of these patients [50].

Although IL-5-targeted biologics represent the first major therapeutic advance in EGPA in decades, both mepolizumab and benralizumab effectively maintain remission and provide significant GC-sparing effects with high complete remission rates. However, the occurrence of EGPA in patients receiving anti-IL-5 therapy for asthma suggests that these agents may not fully prevent disease onset, underscoring the need to better define their positioning in treatment algorithms. Sequential or combination biologic strategies are currently under investigation in EGPA and other diseases. Ultimately, treatment selection should be individualized based on patient preference, adherence, and the presence of coexisting type 2 inflammatory conditions.

#### 6.2.3. Comparison Between Rituximab and Cyclophosphamide

Rituximab is a chimeric monoclonal antibody targeting the CD20 antigen on B cells. It is approved by the FDA for the induction and maintenance of remission in MPA and GPA. Although not formally approved for EGPA, it is considered an alternative to CTXs, either for remission induction or maintenance [86,105]. The REOVAS trial is the first randomized controlled trial evaluating rituximab in EGPA [106]. This study compared glucocorticoids plus rituximab (1 g administered two weeks apart) with a conventional strategy (GCs alone or combined with cyclophosphamide in severe disease) in 105 patients with active disease (BVAS ≥ 3). No statistically significant differences were observed between groups in the primary endpoint (remission defined as BVAS = 0 and prednisone ≤ 7.5 mg/day at day 180) or in secondary endpoints, including duration of remission, relapse rates, severe relapse rates, mean daily GC dose, and adverse events. Specifically, 63.5% of patients in the rituximab group achieved the primary endpoint compared with 60.4% in the control group (relative risk 1.05, 95% CI 0.78–1.42; *p* = 0.75). The mean duration of remission was 48.5 ± 6.5 weeks in the rituximab group versus 49.1 ± 7.4 weeks in the conventional therapy group (*p* = 0.41) [106]. However, long-term follow-up suggested a trend toward lower relapse rates in ANCA-positive patients (specifically MPO-ANCA-positive) treated with rituximab. Although adding rituximab to standard GC therapy did not confer a significant advantage in non-severe EGPA, the study design did not allow a direct equivalence comparison between rituximab and cyclophosphamide.

#### 6.2.4. Repulse Predictors

Relapse of EGPA is defined as the recurrence of clinical signs or symptoms attributable to active disease following a period of remission [3]. The reported relapse rate ranges from 25% to 49% [107,108]. The 2023 EGPA guideline [3] emphasizes that when defining relapse, it is recommended to distinguish between relapse of systemic vasculitis and isolated exacerbations of asthma and ENT manifestations. An increase in eosinophil count without accompanying clinical symptoms should not be considered a relapse [3]. Treatment of relapse depends on its type, clinical severity, prior treatments, and the burden of chronic damage [3]. Currently, no reliable biomarkers are available to measure disease activity or predict relapse in EGPA. However, some studies have linked a higher relapse risk to a peripheral blood eosinophil count (BEC) of less than 3000 cells/μL at diagnosis, cutaneous manifestations, and positive MPO-ANCA [109,110]. In a cohort study involving 141 patients [111], eosinophil count, ESR, CRP, and IgE concentrations showed weak or no association with disease activity and disease flares, suggesting that the role of these parameters as longitudinal biomarkers is limited. Furthermore, the value of serum IgG4 concentration as a biomarker of disease activity remains controversial. Although an observational study including 72 patients with AAV [112] found that serum IgG4 concentrations were markedly increased in patients with active EGPA and correlated positively with BVAS and the number of organs involved, these data have not yet been confirmed. The utility of ANCA monitoring in EGPA is also debated [91], as ANCA positivity or titers are not clearly associated with disease activity or treatment response. However, the persistence, rise, or reappearance of ANCA may be a useful indicator prompting more intensive clinical assessment [113].

#### 6.2.5. Long-Term Outcomes and Prognosis

Owing to the widespread availability of ANCA testing, early diagnosis, novel therapeutic agents, and the effectiveness of immunotherapy, survival in AAV has improved significantly over the past decade, reaching 70–80% [114]. Nevertheless, mortality resulting from complications of either the disease itself or its treatment remains higher than anticipated. The FFS serves as a prognostic tool, and a score exceeding 2 is associated with poorer outcomes at five years [115]. With respect to prognosis, substantial heterogeneity exists in EGPA regarding age at onset, pattern of organ involvement, disease severity, treatment response, and risk of comorbidities, all of which influence individual outcomes. Advances in treatment regimens have, to a certain extent, improved survival in EGPA. Consequently, the focus of clinical management has shifted toward long-term health maintenance, prevention of disease flares, minimization of treatment-related toxicity, and improvement of quality of life.

### 6.3. Follow-Up

Reliable biomarkers for long-term monitoring of EGPA are currently lacking. A 2023 guideline on EGPA management recommends regular monitoring of relevant clinical manifestations, particularly pulmonary function, cardiovascular events, and neurological complications [3]. For EGPA patients followed in the Allergy department, follow-up should focus on PFTs, FeNO, and chest/sinus CT changes. If extra-pulmonary organ involvement emerges or worsens, prompt referral for multidisciplinary consultation is advised to optimize management.

## 7. Future Perspectives

### 7.1. Multidisciplinary Management and Collaboration

Multidisciplinary management in centres with expertise in vasculitis is considered an overarching principle in the treatment guidelines for AAV [74]. Furthermore, the diagnostic evaluation of patients with suspected EGPA should always be multidisciplinary [3], although EGPA may initially present to allergy departments owing to its mimicking of allergic manifestations. Some retrospective studies have demonstrated that centre-based management improves patient outcomes [116,117]. In addition, collaborative efforts among international research centres, clinical registries, and patient populations have the potential to expand knowledge regarding the pathogenesis and management of EGPA, and may facilitate the identification of novel biomarkers, the development of treatment strategies, and the formulation of clinical guidelines.

### 7.2. An Integrated View of Disease Activity, Organ Damage, and PROs

Although standardised tools such as the BVAS and the AAV-PRO questionnaire have improved the ability to assess disease activity, damage, and health-related quality of life (HRQoL), integrating these tools into routine care to evaluate disease activity and damage has proven challenging, particularly in patients with EGPA. This difficulty arises because the assessment of disease activity and damage in EGPA requires more precise incorporation of ENT manifestations and asthma [74]. Therefore, future research may need to focus on combining disease assessment tools with patient-reported outcomes and biomarkers to better characterise the disease and evaluate outcomes in EGPA.

### 7.3. Composite Outcomes for Prognostic Prediction

No composite assessment tool specifically designed for EGPA is currently available. However, the Outcome Measures in Rheumatology (OMERACT) Vasculitis Working Group is developing a composite tool that captures the full disease burden across multiple domains, aiming to provide a more comprehensive assessment of AAV. This tool may guide clinical trials and patient management in the future, and has the potential to incorporate patient-reported outcomes, biomarkers, and genetic data [118,119].

## 8. Conclusions

EGPA is a complex, multisystem disease that often presents initially with asthma, rhinitis, and sinusitis, making the Allergy department a critical entry point for early diagnosis. Given the lack of definitive diagnostic biomarkers, early recognition relies heavily on clinical suspicion and systematic evaluation of eosinophilia, ANCA status, and multi-organ involvement. A structured diagnostic and treatment pathway, as proposed in this article, can help allergists identify EGPA at an earlier stage, potentially reducing diagnostic delays and improving long-term outcomes. Current treatment strategies are stratified by disease severity and include glucocorticoids, conventional immunosuppressants, and increasingly, targeted biologic therapies such as anti-IL-5 agents and rituximab. Despite therapeutic advances, challenges remain in predicting relapse, monitoring disease activity, and preventing treatment-related toxicity. Future efforts should focus on integrating multidisciplinary care, patient-reported outcomes, and emerging biomarkers to further personalize and optimize EGPA management.

## Figures and Tables

**Table 1:** Classification Criteria for EGPA.

1990 ACR Classification Criteria	2022 ACR/EULAR Classification Criteria
1. Asthma	Eosinophil count ≥ 1.0 × 10^9^/L (+5)
2. Eosinophilia > 10% or >1.5 × 10^9^/L	Obstructive airway disease (+3)
3. Mononeuropathy or polyneuropathy	Nasal polyps (+3)
4. Non-fixed pulmonary infiltrates	Extravascular eosinophil-predominant inflammation (+2)
5. Paranasal sinus abnormality	Mononeuritis multiplex and/or motor neuropathy not due to radiculopathy (+1)
6. Extravascular eosinophils on biopsy	c-ANCA or anti-PR3 antibody positivity (−3)
	Microscopic hematuria (−1)
**A patient** **can be classified as having EGPA if ≥4 of the 6 criteria are met, after exclusion of other vasculitides.**	**A total score ≥ 6 points suggests classification as EGPA.**

ACR: American College of Rheumatology; EULAR: European Alliance of Associations for Rheumatology; EGPA: Eosinophilic granulomatosis with polyangiitis; ANCA: anti-neutrophil cytoplasmic antibody; PR3: Proteinase 3.

**Table 2:** Differentiation of EGPA from other AAV.

Types	EGPA	GPA	MPA	PAN
**Serology**				
Blood Eosinophils	++++	+	−	−
ANCA	MPO (30–40%)	PR3 (80–95%)	MPO (70–80%)	−
**Clinical Manifestations**				
ENT	79–90%	80–93%	−	−
Respiratory	38–77%	53–83%	25–55%	−
Cardiac	11–76%	4–40%	10–21%	−
Gastrointestinal	20–78%	11–24%	30–58%	Common
Skin	23–68%	33–45%	30–60%	Common
Nervous System	42–74%	20–50%	37–72%	Common
Renal	16–35%	50–80%	80–100%	Uncommon
Ocular	<5%	28–50%	<5%	−
**Histology**				
Granulomas	++++ (Eosinophil-rich)	++++ (Neutrophil-rich)	−	−

AAV: ANCA-associated vasculitis; EGPA: Eosinophilic granulomatosis with polyangiitis; GPA: Granulomatosis with polyangiitis; MPA: Microscopic polyangiitis; PAN: Polyarteritis nodosa; ENT: Ear, Nose, Throat; MPO: Myeloperoxidase; PR3: Proteinase 3. ++++: ≥75%~100%, +: 0~<25%; −: negative.

**Table 3:** Treatment Principles for Induction of Remission, Maintenance Therapy, and Relapse in EGPA.

Disease Severity	Induction Therapy	Maintenance Therapy	Relapse Therapy
**Active Severe EGPA**	GC IV pulses or high-dose oral GC + CTX or RTX. Cardiac involvement, ANCA-negative with severe neuropathy/GI involvement, preferred CTX. ANCA-positive, active glomerulonephritis, prior CTX exposure, fertility concerns, preferred RTX.	MTX/AZA/MMF	Recommend re-induction with RTX. Relapse with cardiac involvement or rapid relapse post-RTX; consider CTX.
**Active Non-Severe EGPA**	First-line: Mepolizumab + GC, Second-line: MTX/AZA/MMF + GC.		Mepolizumab

EGPA: Eosinophilic granulomatosis with polyangiitis; GC: Glucocorticoids; CTX: Cyclophosphamide; RTX: Rituximab; MTX: Methotrexate; AZA: Azathioprine; MMF: Mycophenolate mofetil.

## Data Availability

No new data were created or analyzed in this study. Data sharing is not applicable to this article.
